# Supplementary material for: Complexome profiling of the Chlamydomonas psb28 mutant reveals TEF5 as an early PSII assembly factor
Source: Plant Cell. 2025 Mar 17;37(6):koaf055. doi: 10.1093/plcell/koaf055 (PMC12164590; doi:10.1093/plcell/koaf055)
Supplement: koaf055_Supplementary_Data [file koaf055_supplementary_data.zip › psb28_interactive.zip!/plots/intensity/qualityPlots/peptides/PRPL1_Cre02.g088900.t1.2.html]

### Cre02.g088900.t1.2

Trivial name: PRPL1  
SeqCoverage [median]: 24.90  
Mass: 32.44 kDa  
  
 

| Sequence | MissedCleavage | Mass [Da] | wtIntensity | muIntensity | Score |
| --- | --- | --- | --- | --- | --- |
| AGTVTTDVAGTVK | 0 | 1218.65 | 0.00 | 1535.16 | 110.39 |
| AVQESIDANKPPGAK | 1 | 1523.79 | 1707.51 | 0.00 | 46.48 |
| DAGADVVGGDDLIEK | 0 | 1472.70 | 410.61 | 2891.59 | 107.09 |
| FTESVEFHAR | 0 | 1221.58 | 0.00 | 352.06 | 69.20 |
| IGVSALQNTK | 0 | 1029.58 | 1957.90 | 2625.45 | 159.79 |
| VAVLTQNDNIK | 0 | 1213.67 | 577.17 | 1390.74 | 109.44 |
|  |  |  | 4653.2 | 8795.0 |  |
